# A visual approach towards forward collision warning for autonomous vehicles on Malaysian public roads

**DOI:** 10.12688/f1000research.72897.1

**Published:** 2021-09-16

**Authors:** Man Kiat Wong, Tee Connie, Michael Kah Ong Goh, Li Pei Wong, Pin Shen Teh, Ai Ling Choo

**Affiliations:** 1 Faculty of Information Science and Technology, Multimedia University, Melaka, Melaka, 75450, Malaysia; 2 School of Computer Sciences, Universiti Sains Malaysia, Penang, Penang, 11800, Malaysia; 3 Department of Operations, Technology, Events and Hospitality Management, Faculty of Business and Law, Manchester Metropolitan University, Manchester, Manchester, M15 6BH, UK; 4 iRadar Sdn. Bhd., Melaka, Melaka, 75450, Malaysia

**Keywords:** Object recognition, Forward Collision Warning, Lane detection, Autonomous vehicles, Computer Vision

## Abstract

**Background:** Autonomous vehicles are important in smart transportation. Although exciting progress has been made, it remains challenging to design a safety mechanism for autonomous vehicles despite uncertainties and obstacles that occur dynamically on the road. Collision detection and avoidance are indispensable for a reliable decision-making module in autonomous driving.

**Methods: **This study presents a robust approach for forward collision warning using vision data for autonomous vehicles on Malaysian public roads. The proposed architecture combines environment perception and lane localization to define a safe driving region for the ego vehicle. If potential risks are detected in the safe driving region, a warning will be triggered. The early warning is important to help avoid rear-end collision. Besides, an adaptive lane localization method that considers geometrical structure of the road is presented to deal with different road types.

**Results:** Precision scores of mean average precision (mAP) 0.5, mAP 0.95 and recall of 0.14, 0.06979 and 0.6356 were found in this study.

**Conclusions: **Experimental results have validated the effectiveness of the proposed approach under different lighting and environmental conditions.

## Introduction

Road traffic accidents are one of the major causes of death in the world. According to a study by the World Health Organization, approximately 1.35 million people die each year due to road traffic injuries. ^1^ In fact, road traffic injuries have become the fifth leading cause of death worldwide. Along this line, the autonomous vehicle has shown to be one of the promising technologies to reduce traffic crashes, especially those caused by human error. ^2^

Autonomous vehicles, or sometimes called advanced driver-assistance systems, are inventions that aim to improve a vehicle’s safety. ^3^ An autonomous vehicle is capable of operating without human control, and decisions can be made independently by the intelligent control system.

The development of autonomous vehicles is still faced with a number of challenges due to the complex and dynamic driving environment. In this paper, a vision-based forward collision warning method is presented. The proposed method monitors the roadway ahead and warns the driver when a risk for collision is detected in a predefined driving region. The proposed forward collision warning architecture is made up of two components: (1) Environment perception, and (2) Lane localization. The environment perception module is used to observe the surrounding of the ego vehicle based on visual input. The lane detection component is responsible to track the reference lane markers ahead of the vehicle. Then a safe driving region is determined by integrating the output of the two modules. If an obstacle is detected in the safe driving region, a warning will be triggered. The proposed approach avoids rear-end collisions by issuing early warnings.

The contributions of this paper are twofold: first, a robust forward collision warning architecture that combines environment perception and lane localization techniques are introduced. Second, an adaptive sliding window approach is proposed to detect potential lane markers on different road conditions. The proposed approach checks the confidence level of the road sign markers in each window and adaptively spawns new neighboring windows to cope with lane lines that deviates from the norm.

## Methods

### Ethics statement

This work has been approved by MMU Research Ethics Committee (Approval number: EA1432021).

### Environment perception

In this paper, the YOLO v5 architecture ^3^ is adopted to detect vehicles and other objects around the ego vehicle. YOLOv5 is selected due to its appealing performance in real-time performance. The mosaic data augmentation strategy employed in its architecture greatly improves the accuracy and robustness of object detection. ^3^ Most importantly, YOLOv5 is lightweight in size and is very fast, making it suitable for a real-time application like autonomous driving.

### Lane localization

Segmenting lane markers from the image is crucial in lane detection. Different combinations of gradients and perceptual spaces are explored to differentiate lane markers from the road surface.

*Color-based feature extraction*

Both the RGB (red, green, blue) color space and HLS (hue, saturation, lightness) color space are investigated. The RGB color space is a common model to represent the three primary colors. The HLS color space, on the other hand, constitutes components that are more closely aligned to human perception. ^4^ Let *R*, *G* and *B* represent the red, green and blue components in a road surface image, the transformation to the HSL model can be achieved by, ^4^

$$H=\arctan\left(\frac{\sqrt{3}\left(G-B\right)}{\left(R-G\right)+\left(R-B\right)}\right)$$ (2)

$$L=\frac{\left(R+G+B\right)}{3}$$ (3)

$$S=1-\frac{\min\left(R,G,B\right)}{L}$$ (4)

**Figure 1.**
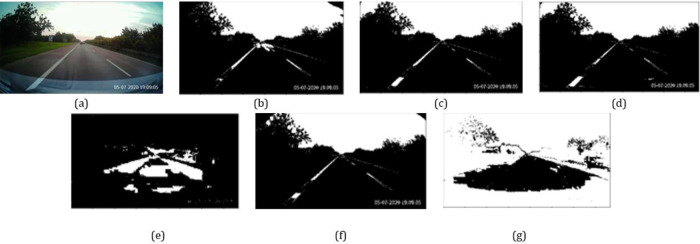
(a) Original image; (b)-(d) Thresholded regions from *R*, *G* and *B* components; (e)-(g) Thresholded regions from *H*, *L* and *S* components.

A pixel in the image is considered the region containing the lane markers if it exceeds some threshold values for each respective color component. Figure 1 depicts some sample threshold regions for the different color dimensions. The Otsu thresholding technique ^5^ is applied. It can be observed that the three primary color components, *R*, *G* and *B*, as well as the lightness attribute, *L*, are able to highlight the lane markers in the image.

*Gradient-based feature extraction*

The Sobel gradient operator ^6^ is used to approximate the image gradient with respect to the horizontal and vertical directions. Given a grayscale version of a road surface image *M*, the gradient of the image in the horizontal, $M_{h}$ , and vertical directions, $M_{v}$ , are computed as,

$$M_{h}=\left[\begin{array}{ccc} -1 & -2 & -1\\ 0 & 0 & 0\\ 1 & 2 & 1 \end{array}\right],\;M_{v}=\left[\begin{array}{ccc} -1 & 0 & 1\\ -2 & 0 & 2\\ -1 & 0 & 1 \end{array}\right]$$ (5)

The gradient magnitude is found by,

$$\left\|M\right\|=\sqrt{M_{h}^{2}+M_{v}^{2}}$$ (6)

**Figure 2.**
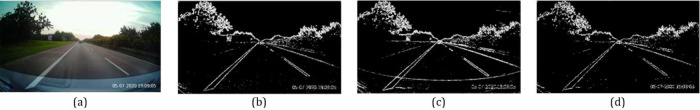
(a) Original image, (b), (c) and (d) Threshold results for *M _h_*, *M _v_* and ‖ *M*‖.

A pixel in $\left\|M\right\|$ is considered a candidate for the lane markers if $\left\|M\right\|\geq T$ for some threshold value *T.* In this study, Otsu thresholding is used to find *T.* Some sample threshold results for $M_{h}$ , $M_{v}$ , and $\left\|M\right\|$ are shown in Figure 2.

*Features fusion*

**Figure 3.**
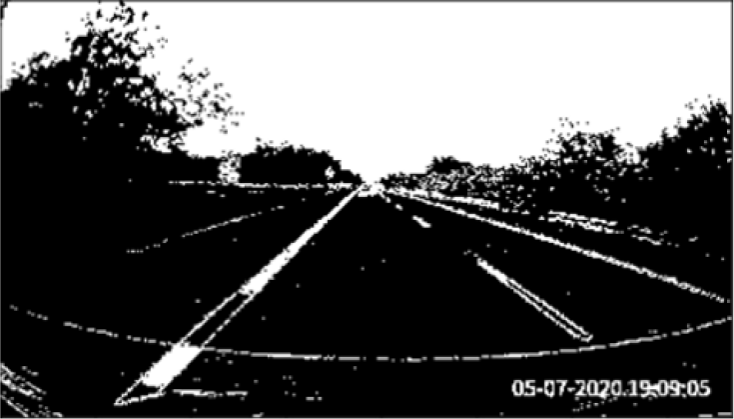
Final output, *F.*

Five features are selected to form the final representation, *F*, for the lane markers image. The selected features are $M_{h}$ , $M_{v}$ , $\left\|M\right\|$ , *L* and *G.* It is obvious that the lane markers can be highlighted with the gradient features. So all the gradient features are selected. The lighting component, *L*, is effective against illumination changes so this feature is also chosen. As the road markers can be distinguished well in all of the color dimensions, the *G* component is empirically selected. The color- and gradient-based features are then fused to form, *F*, using majority voting as,

$$r\left(x,y\right)=\text{mode}\left\{M_{h}\left(x,y\right),M_{v}\left(x,y\right),\left\|M\right\|\left(x,y\right),G\left(x,y\right),L\left(x,y\right)\right\}$$ (7)

$$F\left(x,y\right)=\left\{\begin{array}{cc} 1, & \text{if}\;r\left(x,y\right)=1\\ 0, & \text{otherwise}\; \end{array}\right.$$ (8)

where *x* and *y* represent the coordinate of the individual pixel in the image and r signifies the most frequently occurring values based on the mode function. The final output, F , is illustrated in Figure 3. We observe that the line markers can be shown clearly on the road surface.

*Perspective transformation*

**Figure 4.**
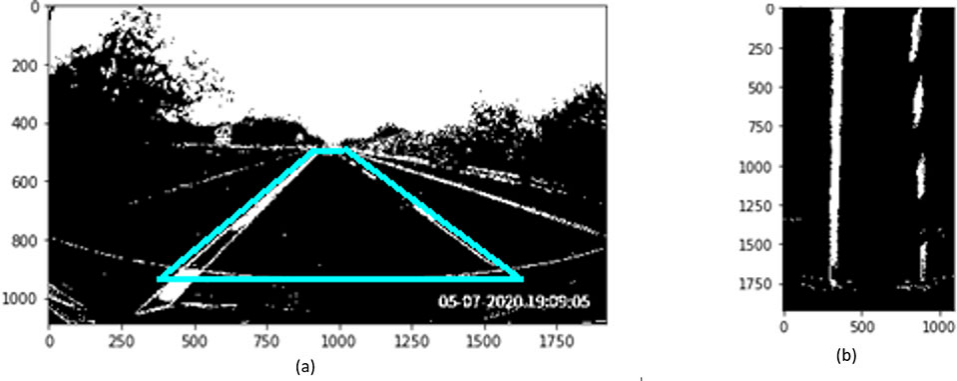
Perspective transformation, (a) Source image in oblique view, (b) Warped result into birds-eye view.

Due to the perspective of a camera mounted on the central region of the ego vehicle’s dashboard when capturing the front view, the lane line segments seem to converge to a point known as the vanishing point problem ^7^ ( Figure 4). Perspective transformation is applied to transform the oblique angle into birds-eye view.

The trapezoidal region in Figure 4a is selected to establish the world of coordinate system for the transformation. Figure 4b illustrates the result after warping the oblique view to aerial view using perspective transformation.

*Sliding window*

**Figure 5.**
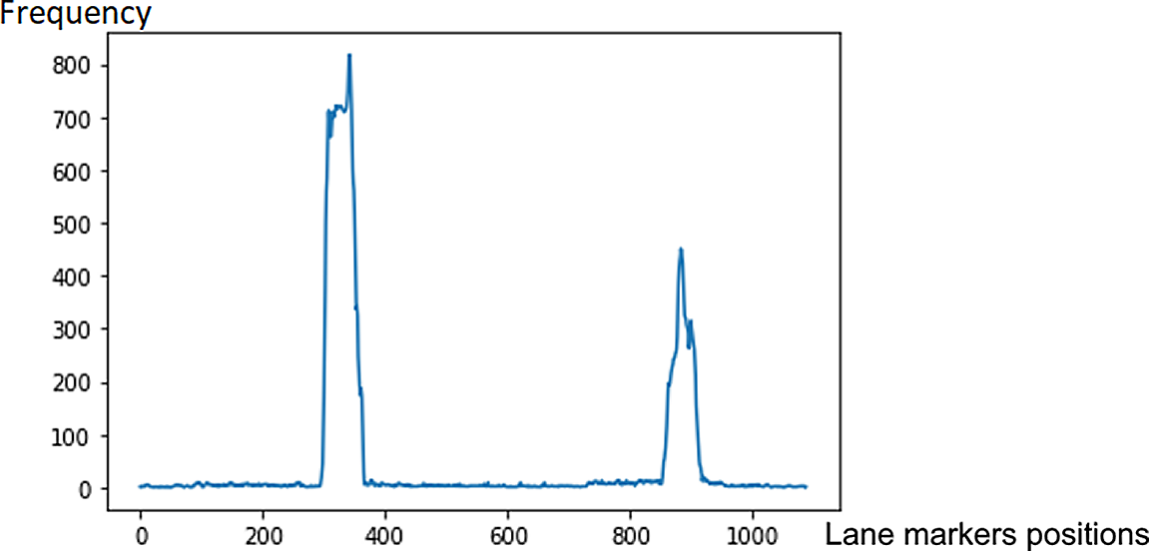
Detecting potential lane markers’ locations based on histogram peaks.

A sliding window approach is applied to detect the lane markers. In Figure 4b, the lane markers appear pretty straight after perspective transformation. We accumulate the pixel values in the vertical direction to detect possible lane marker locations in the image. Locations with the highest number of pixels signifies potential lane markers positions. The histogram for bottom part of Figure 4(b) is presented in Figure 5.

**Figure 6.**
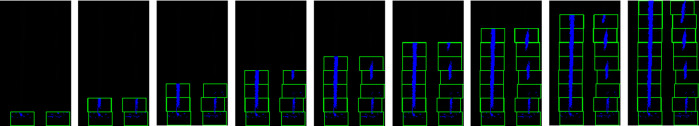
Detecting potential lane markers’ location based on histogram peak.

The peak values locations in the histogram determine the positions to form the initial windows at the bottom of the image (refer Figure 6). The windows locations are determined by the mean of the non-zero pixels values in the windows. Based on these initial windows, another window is drawn as the next sliding window, based on the mean points of the initial windows. The same process is repeated to slide the windows vertically through the image.

The sliding window approach helps to estimate the center of the lane area which is used to approximate lane line curve. However, the algorithm will sometimes lose sight of the lane markers due to broken lines or sharp turning of the road.

**Figure 7.**
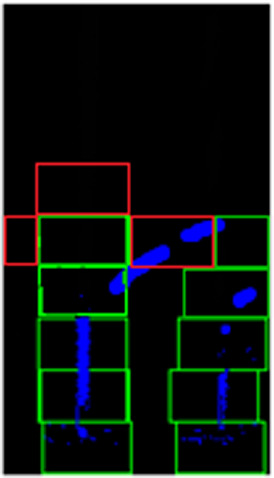
Exploratory windows for non-points regions found on sharp turning.

Therefore, we introduce an adaptive sliding window approach that keeps track of the “strength” of the line markers by checking the number of pixels in a window. The confidence level of the line pixels must exceed a minimum threshold value to qualify the existence of a line. If there is not enough evidence to show the existence of a line in the current window, three exploratory windows will be spawned, i.e. top, left and right, to check the existence of lines in the neighboring regions (refer to the three red windows in Figure 7).

**Figure 8.**
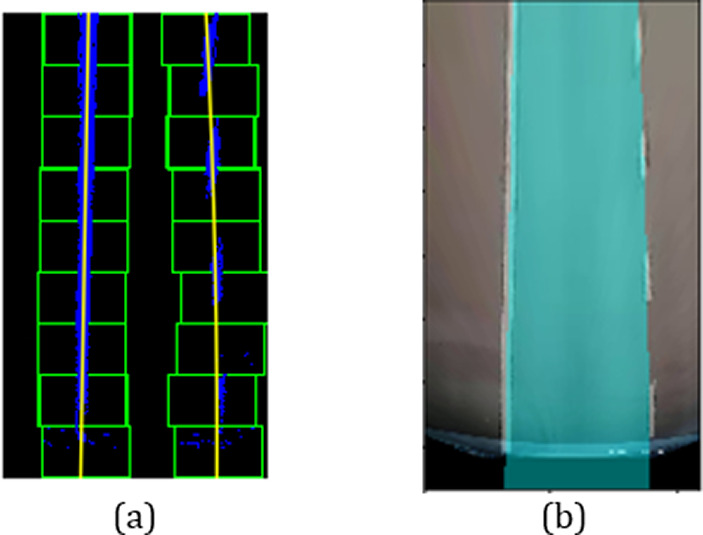
(a) Lane line fitted by polynomial curve, (b) Filled polynomial region.

**Figure 9.**
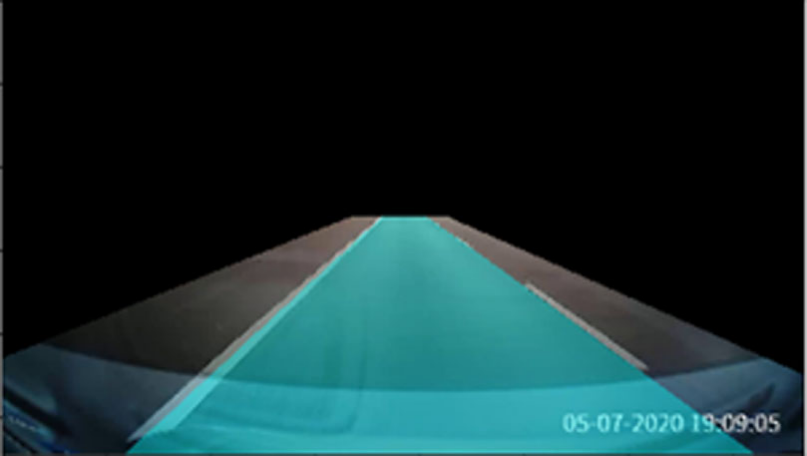
Lane region mapped back to original image.

The points found using the mean values in the sliding windows are used as the control points to approximate the lane line curvature. The third-degree polynomial model ^7^ is used to fit the points on the sliding window as it has simple parameters and has a lower computational cost. Figure 8(a) shows the lane line fitted by the polynomial curve. The fitted region is filled with blue color to highlight the lane region as illustrated in Figure 8(b). Figure 9 depicts the filled lane region that has been warped back to the original perspective view.

### Forward collision warning

*Obstacle detection*

The output of the YOLO algorithm is a tuple containing 5 outputs, $\left(l,\mathrm{bx},\mathrm{by},\mathrm{bw},\mathrm{bh}\right)$ , where l represents the predicted class label, bx , by , bw and bh denote the x and y coordinates and also width and height of the bounding box, respectively. Assume the width and height of the original image are given by *w* and *h*, the location of an object/obstacle detected on the road can be found by,

$$x_{\text{center}}=\mathrm{bx}*w$$ (10)

$$y_{\text{center}}=\mathrm{by}*h$$ (11)

width=bw∗w (12)

height=bh∗h (13)

$$x_{\text{left}}=\frac{\left(\mathrm{bx}-\mathrm{bw}\right)}{2}\times w$$ (14)

$$y_{\mathrm{top}}=\frac{\left(\mathrm{by}-\mathrm{bh}\right)}{2}\times h$$ (15)

where $x_{\text{center}}$ , $y_{\text{center}}$ , width , height , $x_{\text{left}}$ , and $y_{\mathrm{top}}$ are values to calculate the actual bounding box location. Hence, the bounding region of the obstacle detected by YOLO when translated to the image plane, *B*’, can be calculated by $\left[\left(x_{\text{left}}+\text{width},y_{\mathrm{top}}\right),\;\left(x_{\text{left}},y_{\mathrm{top}}\right),\;\left(x_{\text{left}},y_{\mathrm{top}}+\text{heigh}\right),\;\left(x_{\text{left}}+\text{width},y_{\mathrm{top}}+\text{height}\right)\right]$ .

*Warning issuance*

**Figure 10.**
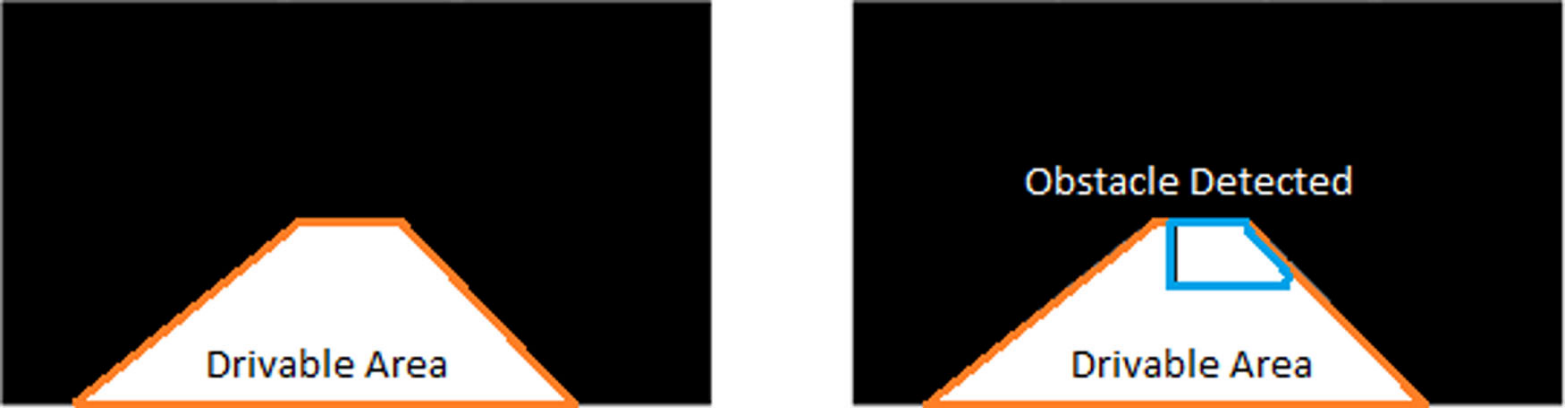
Forward collision warning.

**Figure 11.**
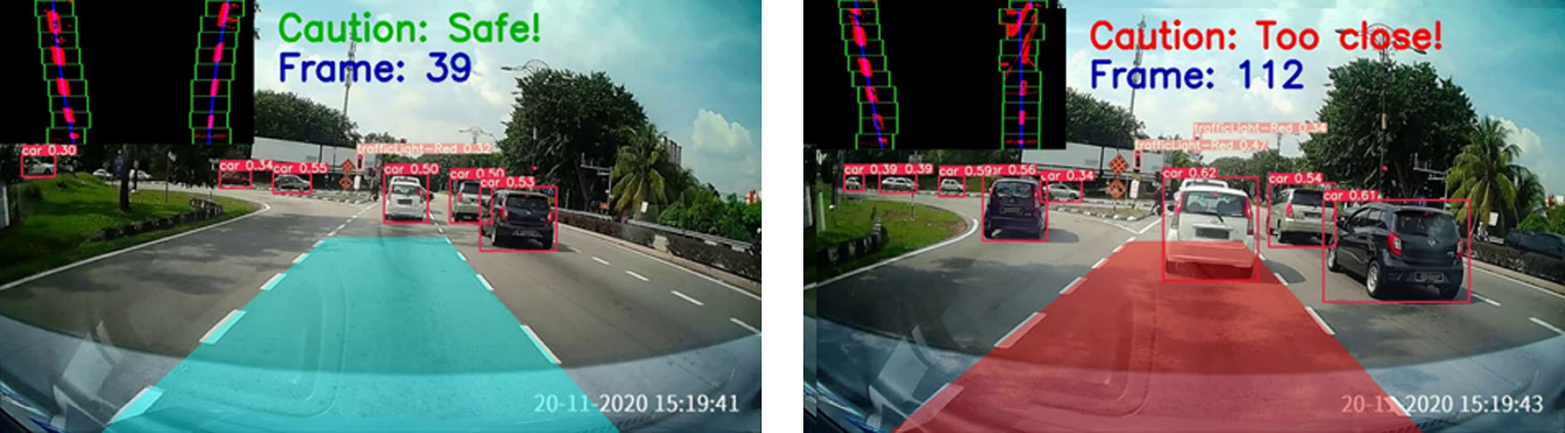
Samples for forward collision warning system.

Given the drivable area, *D*, defined by the polynomial line fit shown in Figure 9, a forward collision warning will be issued if,

$$\text{warning}=\left\{\begin{array}{cc} 1 & D\cap B^{\prime }=0\\ 0 & \text{otherwise} \end{array}\right.$$ (16)

where $B^{\prime }$ refers to the bounding box region for the detected obstacle on the ego lane. Figure 10 displays the safe drivable area (on the left) and an obstacle superimposed on the drivable area (on the right). A warning will be issued in the case when the obstacle is detected on the ego lane drivable area. Some samples of the proposed method are presented in Figure 11.

### Experimental setup and evaluation metrics

All the experiments were conducted on Google Colab with a 1 × Tesla K80 GPU having 2496 CUDA cores, 12GB GDDR5 VRAM, a CPU with a single core hyper threaded Xeon Processors @2.3Ghz (i.e. 1 core, 2 threads), 12.6 GB of RAM and 33 GB of disk.

In this paper, the evaluation metrics used include precision, recall and mean average precision. ^10^ The source code used for the analysis can be found in the *Software availability*. ^12^

### Datasets

The Roboflow Self Driving Car dataset, ^8^ a modified version of Udacity Self Driving Car Dataset, ^9^ is used to train the YOLO model. The dataset contains 97,942 labels across 11 classes and 15,000 images. All the images are down-sampled to 512 × 512 pixels. The annotations have been hand-checked for accuracy. The dataset is split into training set (70%), testing set (20%) and validation set (10%).

The videos/images used to assess the effectiveness of the proposed forward collision warning approach were collected by the authors manually on Malaysian public roads and can be found as *Extended data*. ^11^ A Complementary Metal Oxide Semiconductor (CMOS) camera in a smartphone was used to capture the videos/images of the roads. The camera was placed at the centre of the car’s dashboard using a phone holder. The camera recorded the frontal view of the car while the vehicle moved along the road. The data were recorded on two road types: (1) normal road (i.e. federal roads), and (2) highways. The data were captured during different times of the day, e.g. morning and night. All the images are resized to 512 × 512 pixels.

## Results

### Performance for object detection results

The performance for object detection was evaluated using different combinations of hyperparameters. Different image sizes were tested, ranging from 64 × 64, 288 × 288 to 512 × 512. Two optimizers namely stochastic gradient descent (SGD) and ADAM optimizer were assessed. The batch sizes are searched in the range {16, 32, 64}.

Table 1 presents the performance metrics for the different hyperparameters combinations. ^11^ In the table, mAP 0.5 and mAP 0.95 refer to the mean average over intersection over union (IoU) thresholds of 0.5 and 0.95, respectively. We observe that the SGD optimizer with 64 batch size of 512 × 512 input size yields the highest mAP 0.5, mAP 0.95 and recall. The highest precision score is achieved by the SGD optimizer with 16 batch size on 512 × 512 input size.

**Table 1.** Performance matrix for different hyperparameters. mAP 0.5 and mAP 0.95 refer to the mean average over IoU thresholds of 0.5 and 0.95, respectively.

Model	mAP 0.5	mAP 0.95	Precision	Recall
64_ADAM_16	0.007049	0.002032	0.01368	0.03901
64_ADAM_32	0.006256	0.001722	0.03511	0.03816
64_ADAM_64	0.006088	0.001853	0.008441	0.03395
64_SGD_16	0.007992	0.002626	0.01144	0.0415
64_SGD_32	0.007436	0.002381	0.005875	0.04152
64_SGD_64	0.008219	0.002733	0.0167	0.04576
288_ADAM_16	0.007049	0.002032	0.01368	0.03901
288_ADAM_32	0.006256	0.001722	0.03511	0.03816
288_ADAM_64	0.006088	0.001853	0.008441	0.03395
288_SGD_16	0.007992	0.002626	0.01144	0.0415
288_SGD_32	0.007436	0.002381	0.005875	0.04152
288_SGD_64	0.008219	0.002733	0.0167	0.04576
512_ADAM_16	0.08725	0.03765	0.07352	0.4887
512_ADAM_32	0.08895	0.03865	0.07661	0.4759
512_ADAM_64	0.08164	0.0361	0.07044	0.4458
512_SGD_16	0.1388	0.06909	0.1014	0.6332
512_SGD_32	0.1369	0.06931	0.1034	0.6351
512_SGD_64	0.14	0.06979	0.1028	0.6356

**Figure 12.**
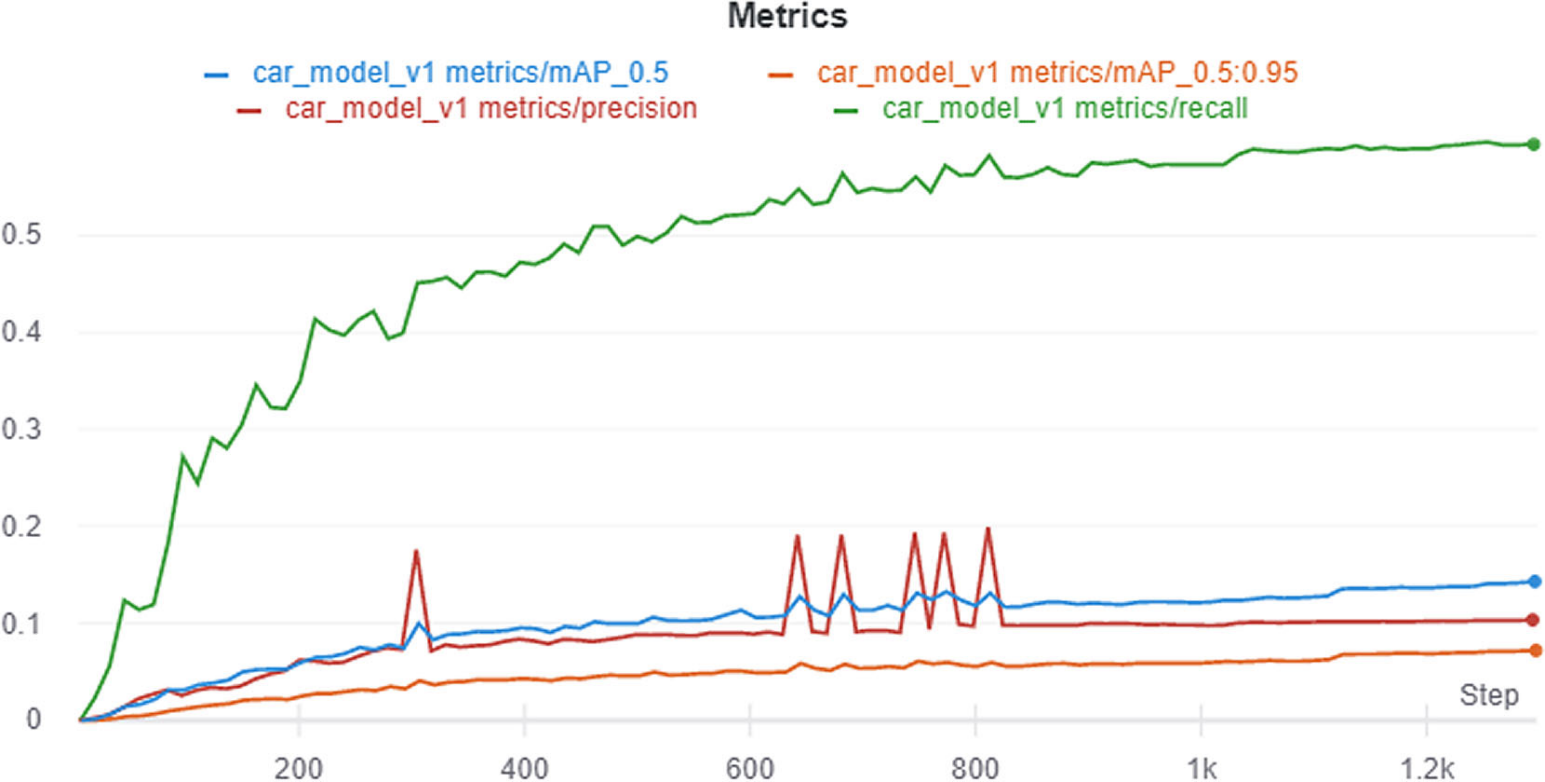
Loses for different hyperparameters.

**Figure 13.**
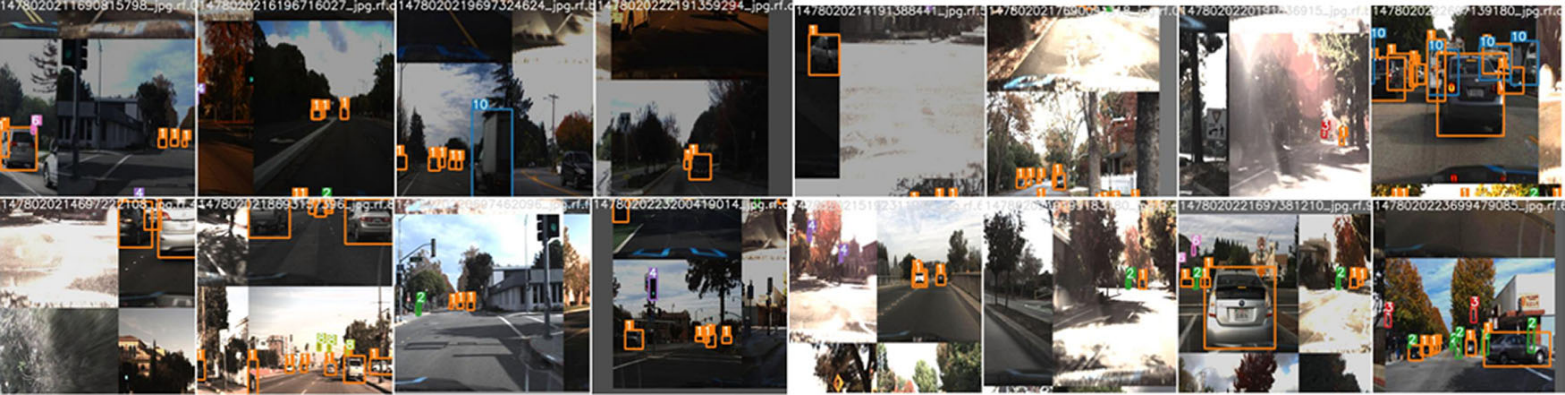
Prediction results for the proposed model.

Overall, the model with SGD optimizer of batch size 64 on 512 × 512 image size yields favorable performance. We name this model car_model_v1. The performance metric after running car_model_v1 for 100 epochs is depicted in Figure 12. Visualization of the prediction results for some randomly chosen samples are shown in Figure 13. The prediction results demonstrate that the model is able to detect the objects satisfactorily.

### Performance of forward collision detection

**Figure 14.**
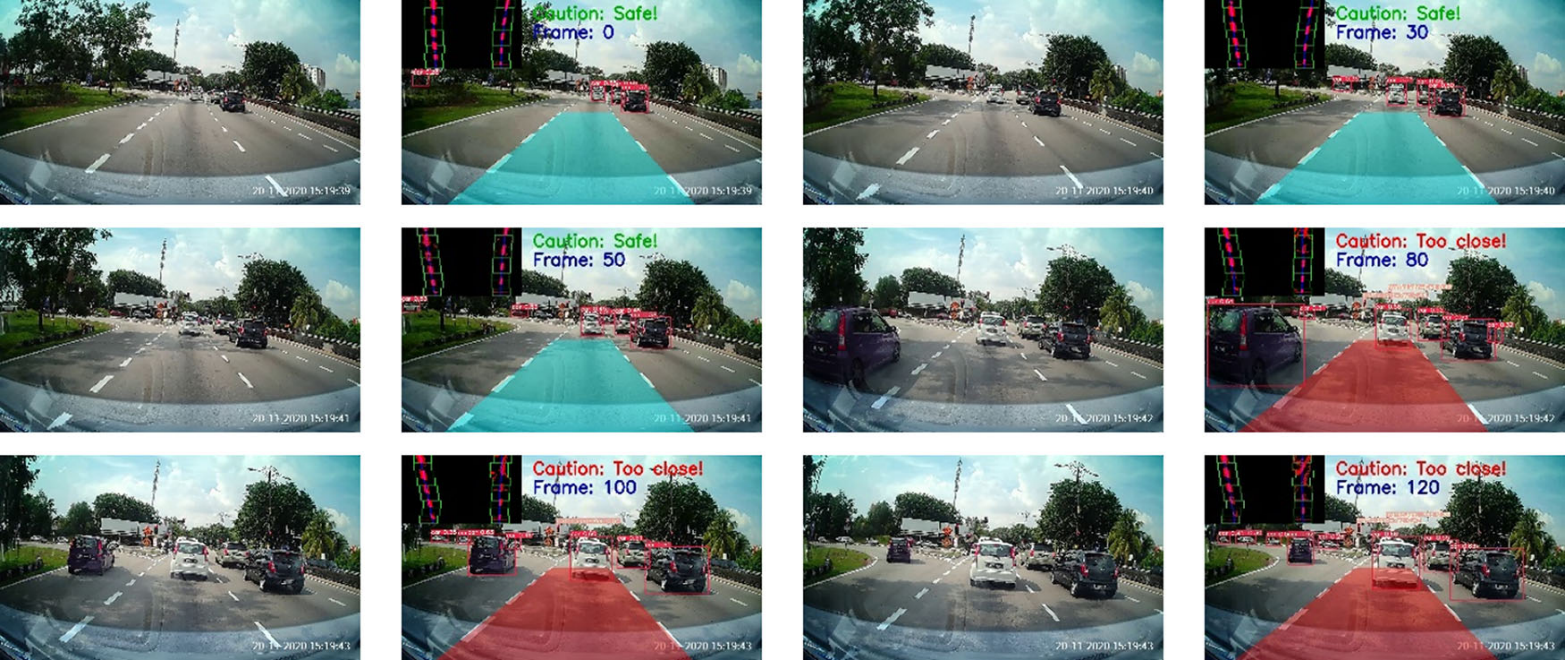
Normal road in the morning.

**Figure 15.**
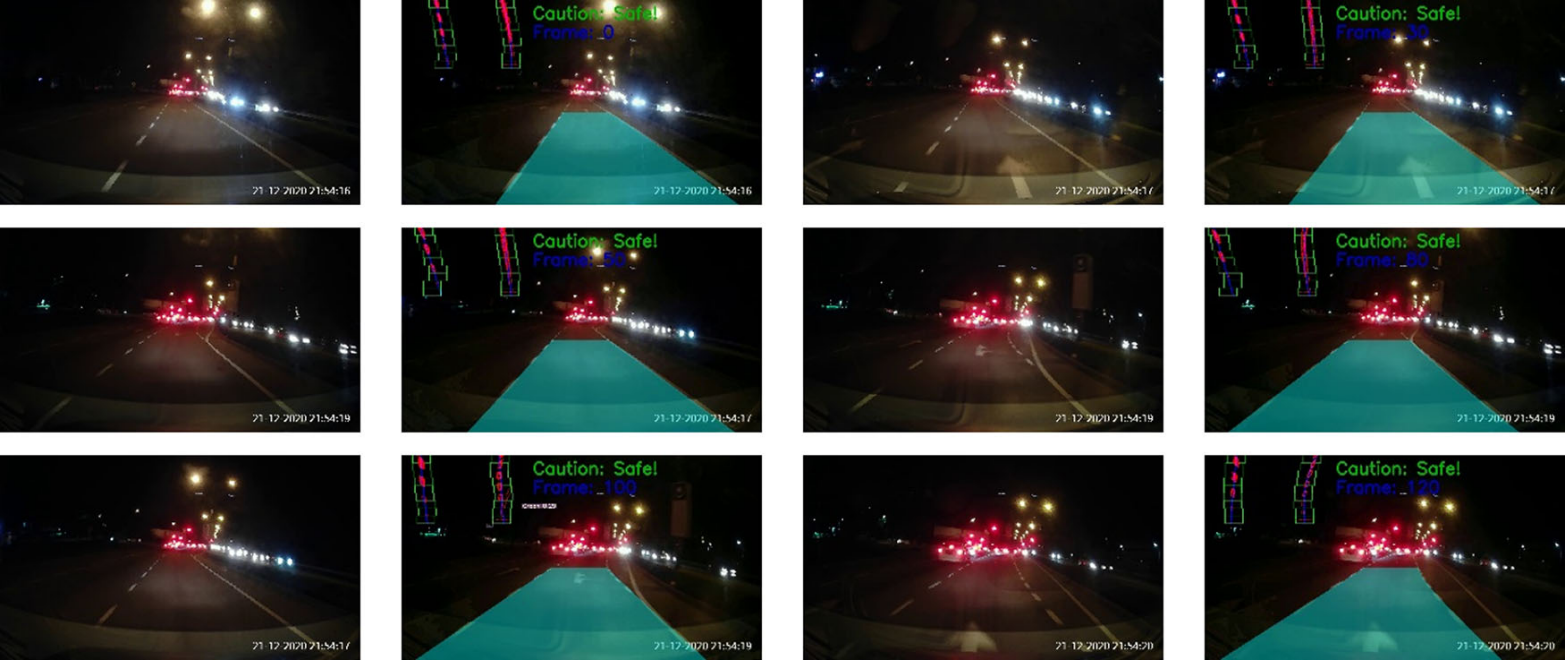
Normal road in the night.

The results of the proposed method for different road conditions are presented in Figures 14 to 15. Figure 14 depicts the testing results on a normal road during the day. The results show a sequence of the ego car moving on the road (from top to bottom, left to right). Initially, there is a safe driving distance between the ego car and the forefront vehicles so the driving region is marked blue. However, as the ego vehicle draws nearer, the vehicle at the front (i.e. the white color car) starts to overlap with the safe driving region. Hence, a warning is triggered and the driving region is marked as red. Another scenario for normal road at night is illustrated in Figure 15. It can be observed that the proposed algorithm also works well during the night in estimating the safe driving region.

**Figure 16.**
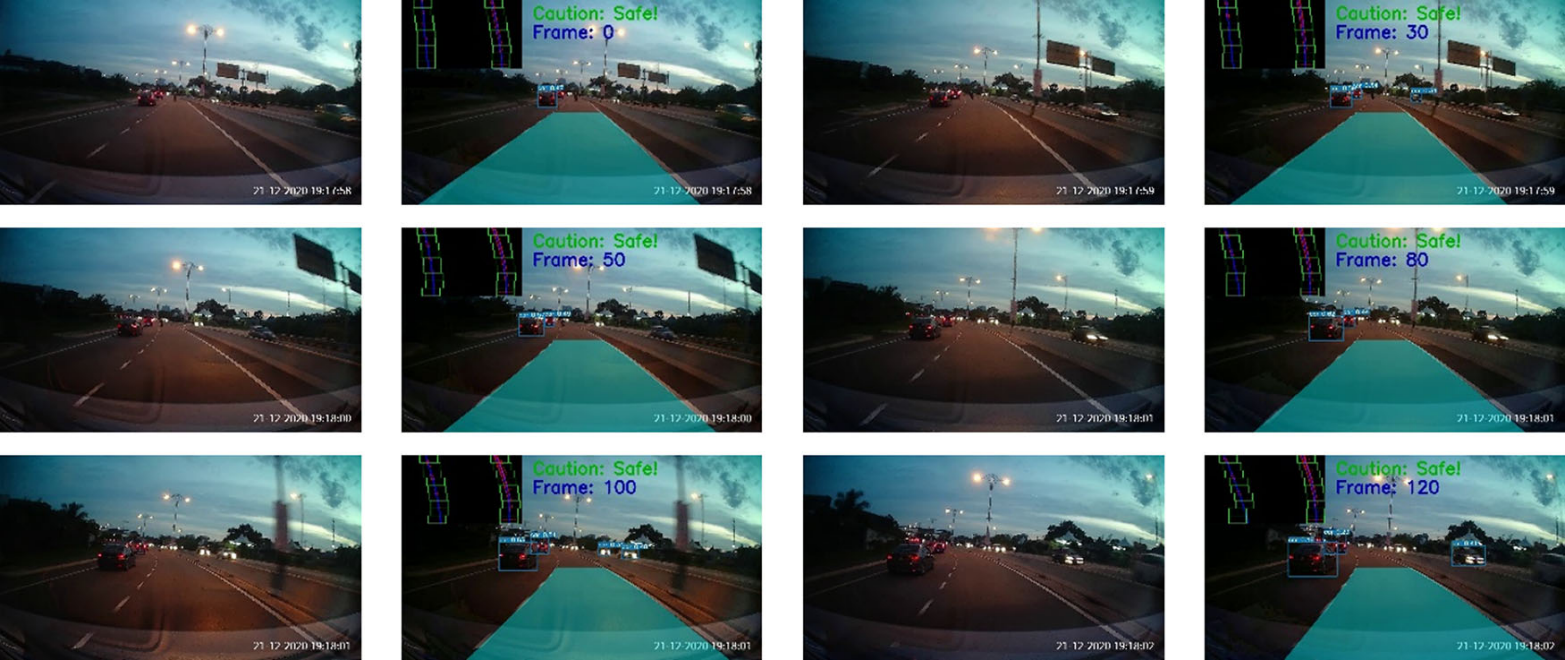
Highway in the morning.

**Figure 17.**
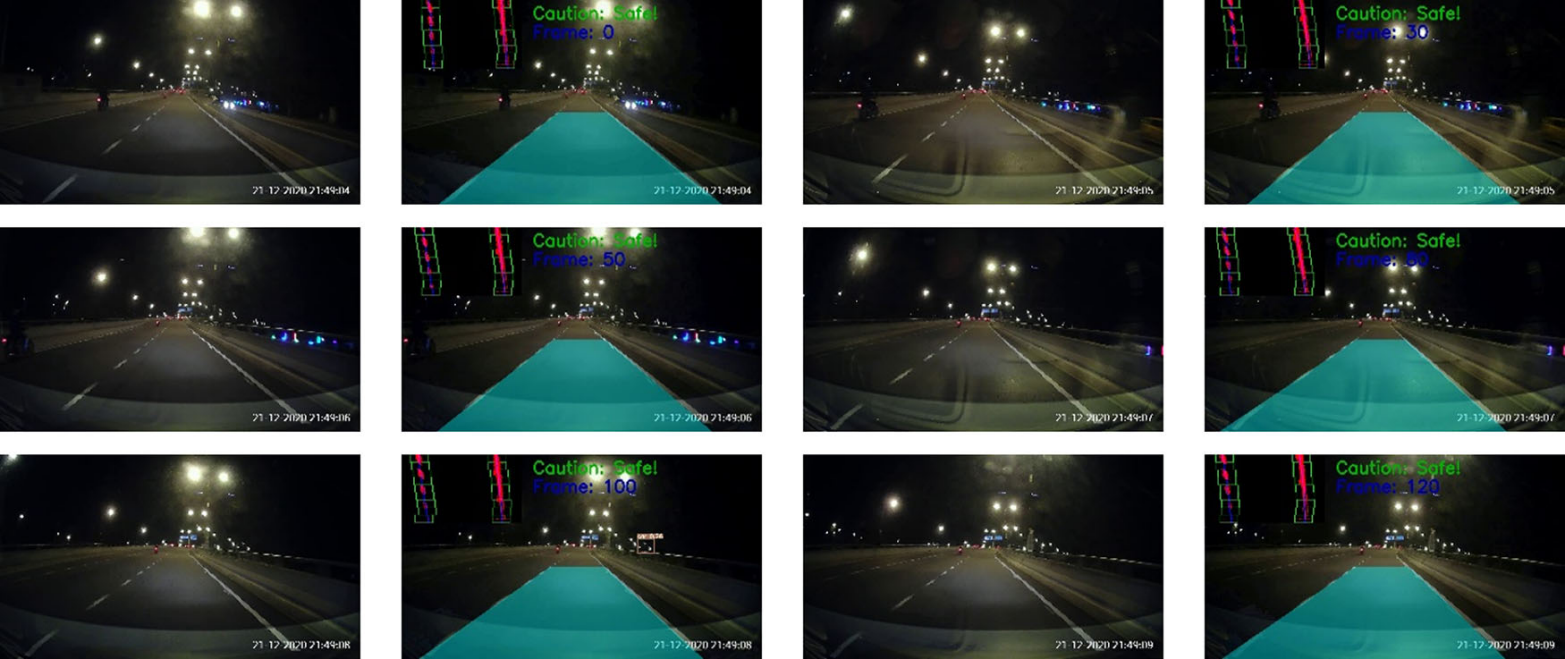
Highway at night.

The tests were also performed on Malaysia highways. The results for morning and night settings are depicted in Figures 16 and 17, respectively. Good tracking results are observed for highways. This is because the road condition of the highways are much better than the normal road. For example, the roads are straight and the lanes are wider. The vehicles are able to keep reasonable distances from each other on the highways.

## Conclusions

This paper proposes an integrated approach for forward collision warning under different driving environments. The proposed approach considers the contextual information around the ego vehicle to derive a safe driving region. A warning will be triggered if a potential obstacle is detected in the driving region. Experimental results demonstrate that proposed approach is able to work with different road conditions. Besides, it has tolerance against illumination changes as it is able to work at different times of the day. In the future, attempts will be made to further improve the speed of the proposed approach. The computation speed for the forward collision warning system must be fast enough to cope with real-time autonomous driving’s requirement.

## Data availability

### Underlying data

The Udacity Self Driving Car Dataset is publicly available at: https://public.roboflow.com/object-detection/self-driving-car. Readers and reviewers can access the data in full by clicking the “fixed-small” or “fixed-large” links provided on the website. The available download formats include JSON, XML, TXT and CSV.

Figshare: Lane Detection. https://doi.org/10.6084/m9.figshare.16557102.v2. ^11^

- highway_morning.MOV
- highway_night.MOV
- normal_morning.MOV
- normal_night.MOV (The videos were taken for normal Malaysian road and highway, both day and night).
- performance_matrix_for_hyperparameter.csv

Data are available under the terms of the Creative Commons Zero “No rights reserved” data waiver (CC0 1.0 Public domain dedication).

## Software availability

Source code available from: https://github.com/gkomix88/LaneDetection/tree/v1.1

Archived source code at time of publication: https://doi.org/10.5281/zenodo.5349280. ^12^

License: Data are available under the terms of the Creative Commons Zero “No rights reserved” data waiver (CC0 1.0 Public domain dedication).
