# Supplementary material for: A visual approach towards forward collision warning for autonomous vehicles on Malaysian public roads
Source: F1000Res. 2022 Mar 7;10:928. Originally published 2021 Sep 16. [Version 2] doi: 10.12688/f1000research.72897.2 (PMC8924939; doi:10.12688/f1000research.72897.2)
Supplement: Peer review history

# Peer review: Reviewer response for version 2

**DOI:** 10.5256/f1000research.121778.r126508

**Published:** 2022-03-15

**Authors:** Furqan Alam

**Affiliations:** 1 Department of Computer Science, King Abdulaziz University, Jeddah, Saudi Arabia

I am satisfied with the changes as per my comments.

Is the rationale for developing the new method (or application) clearly explained?

Yes

Is the description of the method technically sound?

Partly

Are the conclusions about the method and its performance adequately supported by the findings presented in the article?

Partly

If any results are presented, are all the source data underlying the results available to ensure full reproducibility?

Yes

Are sufficient details provided to allow replication of the method development and its use by others?

Partly

Reviewer Expertise:

Machine Learning, Deep Learning, Scene Understanding for Autonomous Vehicles, IoT, Smart Cities, Intelligent Pandemic Response Systems, eLearning, Smart Healthcare Applications

I confirm that I have read this submission and believe that I have an appropriate level of expertise to confirm that it is of an acceptable scientific standard.

# Peer review: Reviewer response for version 2

**DOI:** 10.5256/f1000research.121778.r126507

**Published:** 2022-03-09

**Authors:** Yair Wiseman

**Affiliations:** 1 Computer Science Department, Bar-Ilan University, Ramat Gan, Israel

The authors made a decent effort and the paper is certainly approvable.

Is the rationale for developing the new method (or application) clearly explained?

Partly

Is the description of the method technically sound?

Partly

Are the conclusions about the method and its performance adequately supported by the findings presented in the article?

Yes

If any results are presented, are all the source data underlying the results available to ensure full reproducibility?

Yes

Are sufficient details provided to allow replication of the method development and its use by others?

Partly

Reviewer Expertise:

Embedded Systems, Computational Transportation Science, Intelligent Transportation Systems, Process Scheduling, Hardware-Software Codesign, Memory Management, Asymmetric Operating Systems, Computer Clusters, Autonomous Vehicles, Data Compression, JPEG.

I confirm that I have read this submission and believe that I have an appropriate level of expertise to confirm that it is of an acceptable scientific standard.

# Peer review: Reviewer response for version 1

**DOI:** 10.5256/f1000research.76506.r96560

**Published:** 2022-02-25

**Authors:** Furqan Alam

**Affiliations:** 1 Department of Computer Science, King Abdulaziz University, Jeddah, Saudi Arabia

Comment 1: in the Abstract, if the author could highlight why we need this work, then it will be good.

 Comment 2: it would be a good idea if authors would explicitly state the drawbacks of previous works to prove the worth of this work.

 Comment 3: it will be desirable if author gives some kind of block diagram or flowchart of whole process.

 I approve the paper, but strongly recommend that the author must work on above comments to improve the quality of the paper.

Is the rationale for developing the new method (or application) clearly explained?

Yes

Is the description of the method technically sound?

Partly

Are the conclusions about the method and its performance adequately supported by the findings presented in the article?

Partly

If any results are presented, are all the source data underlying the results available to ensure full reproducibility?

Yes

Are sufficient details provided to allow replication of the method development and its use by others?

Partly

Reviewer Expertise:

Machine Learning, Deep Learning, Scene Understanding for Autonomous Vehicles, IoT, Smart Cities, Intelligent Pandemic Response Systems, eLearning, Smart Healthcare Applications

I confirm that I have read this submission and believe that I have an appropriate level of expertise to confirm that it is of an acceptable scientific standard.

# Response

**Published:** 2022-02-28

**Authors:** Tee Connie

**Affiliations:** Multimedia University, Malaysia

Dear Reviewer,

 Thank you very much for your time and efforts in reviewing our manuscript “A visual approach towards forward collision warning for autonomous vehicles on Malaysian public roads”. The manuscript has been improved based on your valuable comments and suggestions. In this response letter, we list the specific concerns and questions raised by the reviewer and provide our itemized response.

**--**

**Point 1: **In the Abstract, if the author could highlight why we need this work, then it will be good.

**Response 1: **We thank the reviewer for the suggestion. The motivation of this work has been added in the abstract as follows:

*This study presents a robust approach for forward collision warning using vision data for autonomous vehicles on Malaysian public roads. The proposed architecture combines environment perception and lane localization to define a safe driving region for the ego vehicle. If potential risks are detected in the safe driving region, a warning will be triggered. Besides, an adaptive lane localization method that considers geometrical structure of the road is presented to deal with different road types. The proposed forward collision warning mechanism enables early warning which is important to help avoid rear-end collision.*

**Point 2: **It would be a good idea if authors would explicitly state the drawbacks of previous works to prove the worth of this work

**Response 2: **Thank you for the suggestion. In the “Environment Perception” section, we have state the drawbacks of the previous works and highlight the advantage of the proposed method as follows:

*An early collision detection model based on bounding volume hierarchies was presented ^13^. Later on, many bounding box-based methods have been introduced. Different from the previous approaches that rely on geometrical analysis of the objects in the scene, this paper proposes a data-driven approach which is more robust to appearance variations. In YOLOv5, the mosaic data augmentation strategy employed in its architecture greatly improves the accuracy and robustness of object detection. ^3 ^Most importantly, YOLOv5 is lightweight in size and is very fast, making it suitable for a real-time application like autonomous driving.*

**Point 3: **It will be desirable if author gives some kind of block diagram or flowchart of whole process.

**Response 3: **Thank you for the suggestion. The flowchart of the whole process has been added in the manuscript as below: *https://f1000researchdata.s3.amazonaws.com/linked/408176.Connie_Comment_Graph.docx*

# Peer review: Reviewer response for version 1

**DOI:** 10.5256/f1000research.76506.r122291

**Published:** 2022-02-22

**Authors:** Yair Wiseman

**Affiliations:** 1 Computer Science Department, Bar-Ilan University, Ramat Gan, Israel

The paper suggests a technique for forward collision warning for autonomous vehicles employing vision information. The paper focuses on Malaysian public roads.

 The English of the paper must be improved, for example;

 due to its appealing performance in real-time performance ->  due to its appealing real-time performance

 into birds-eye view -> into a birds-eye view

 The histogram for bottom part of Figure 4(b) -> The histogram for the bottom part of Figure 4(b)

 The windows locations -> The window locations

 the non-zero pixels values -> the non-zero pixel values

 The paper discusses driverless vehicles; however, they write "The proposed method monitors the roadway ahead and warns the driver when a risk for collision is detected". So, is there a driver or not?

 The method of collision detection employing bounding boxes was suggested 15 years ago in I. Grinberg and Y. Wiseman (2007). ^1^ I would encourage the authors to cite this paper and explain how their work goes beyond it.

Is the rationale for developing the new method (or application) clearly explained?

Partly

Is the description of the method technically sound?

Partly

Are the conclusions about the method and its performance adequately supported by the findings presented in the article?

Yes

If any results are presented, are all the source data underlying the results available to ensure full reproducibility?

Yes

Are sufficient details provided to allow replication of the method development and its use by others?

Partly

Reviewer Expertise:

Embedded Systems, Computational Transportation Science, Intelligent Transportation Systems, Process Scheduling, Hardware-Software Codesign, Memory Management, Asymmetric Operating Systems, Computer Clusters, Autonomous Vehicles, Data Compression, JPEG.

I confirm that I have read this submission and believe that I have an appropriate level of expertise to confirm that it is of an acceptable scientific standard, however I have significant reservations, as outlined above.

## References

[1] : Scalable parallel collision detection simulation. *SIP '07: Proceedings of the Ninth IASTED International Conference on Signal and Image Processingl* .2007;380-385

# Response

**Published:** 2022-02-24

**Authors:** Tee Connie

**Affiliations:** Multimedia University, Malaysia

Dear Reviewer,

 Thank you very much for your time and efforts in reviewing our manuscript “A visual approach towards forward collision warning for autonomous vehicles on Malaysian public roads”.

 According to your valuable comments and suggestions, the manuscript has been imrpoved. In this response letter, we list the specific concerns and questions raised by the reviewer and provide our itemized response.

**--**

**Point 1: **The paper suggests a technique for forward collision warning for autonomous vehicles employing vision information. The paper focuses on Malaysian public roads. The English of the paper must be improved, for example; due to its appealing performance in real-time performance ->  due to its appealing real-time performance; into birds-eye view -> into a birds-eye view; The histogram for bottom part of Figure 4(b) -> The histogram for the bottom part of Figure 4(b); The windows locations -> The window locations the non-zero pixels values -> the non-zero pixel values.

**Response 1: **We thank the reviewer for the careful review. The mistakes have been corrected in the manuscript. We have also proofread the manuscript carefully to make sure the paper is free from language errors.  

**Point 2: **The paper discusses driverless vehicles; however, they write "The proposed method monitors the roadway ahead and warns the driver when a risk for collision is detected". So, is there a driver or not?

**Response 2: **The reviewer has a sharp observation. Yes, the paper is intended for autonomous vehicle development. Hence, the sentence has been revised to “The proposed method monitors the roadway ahead and issues a warning alert when a risk for collision is detected” in the manuscript.

**Point 3: **The method of collision detection employing bounding boxes was suggested 15 years ago in I. Grinberg and Y. Wiseman (2007).1 I would encourage the authors to cite this paper and explain how their work goes beyond it.

**Response 3: **Thank you for the suggestion. The reference has been added. An explanation how the proposed study goes beyond the work has also be provided in the “Environment Perception” section as below:

*An early collision detection model based on bounding volume hierarchies was presented 13. Later on, many bounding box-based methods have been introduced. Different from the previous approaches that rely on geometrical analysis of the objects in the scene, this paper proposes a data-driven approach. In YOLOv5, the mosaic data augmentation strategy employed in its architecture greatly improves the accuracy and robustness of object detection. 3 Most importantly, YOLOv5 is lightweight in size and is very fast, making it suitable for a real-time application like autonomous driving.*
